# Oligopoly competition between satellite constellations will reduce economic welfare from orbit use

**DOI:** 10.1073/pnas.2221343120

**Published:** 2023-10-16

**Authors:** Julien Guyot, Akhil Rao, Sébastien Rouillon

**Affiliations:** ^a^Bordeaux School of Economics UMR 6060, Bordeaux University, Avenue Duguit, 33608 Pessac cedex, France; ^b^Department of Economics, Middlebury College, Middlebury, VT 05753

**Keywords:** space economics, satellites, game theory, oligopoly, common-pool resources

## Abstract

Orbital space enables many essential services, such as weather forecasting, global communication, navigation, Earth observation for environmental and agricultural management, and national security applications. Orbit use is increasingly defined by firms launching coordinated fleets—“constellations”—of satellites into low-Earth orbit. These firms operate in markets with few or no competitors, such as the market for broadband internet provision to rural areas. How will oligopolistic competition shape the allocation of orbital space? We analyze orbital-use patterns and economic welfare when two profit-maximizing firms operate satellite constellations with sophisticated collision avoidance systems. We compare this duopoly equilibrium to public utility constellations designed and regulated to maximize economic welfare from orbit use. We show that imperfect competition reduces economic welfare from orbit use by up to 12%—$1.1 billion USD—per year and distorts the allocation of orbital space. The nature of the distortion depends on the magnitude of constellation-related environmental damages. When damages are low, economic welfare is maximized by larger-than-equilibrium constellations. When damages are high, economic welfare is maximized by smaller-than-equilibrium constellations. Between the growing commercial and national interests in outer space and the importance of low-Earth orbit to space exploration, orbit-use management is likely to be a fruitful and policy-relevant area for economic research. We conclude with a discussion of future research directions in orbit-use management relevant to policymakers around the world.

## Significance

Orbital space is rapidly concentrating among a few commercial operators who manage large coordinated fleets of satellites. Managing these fleets safely requires conducting a high number of collision avoidance maneuvers. Proposed management strategies for these systems have been primarily technological, with less attention to the impacts of economic competition on orbit use. Using a coupled physicoeconomic model, we show that imperfect competition between satellite operators will reduce economic welfare and distort orbital-use patterns relative to optimal public utility systems. These results highlight the need for regulatory policies promoting efficient orbit use in the public interest.

The number of satellites orbiting the earth has grown exponentially in the past decade—nearly half of all objects humanity had launched to space by 2021 were launched between 2011 and 2021 (1, 2). Of these recent objects, more than half are part of a coordinated fleet belonging to a single commercial entity (2, 3). More such commercial low-Earth orbit (LEO) “megaconstellations”—constellations with hundreds or thousands of satellites—are planned or in development, with tens of thousands of new commercial satellites projected to be in orbit in the next decade. The majority of these systems are intended to provide global telecommunications services to populations that are not well served by terrestrial telecommunications providers.

The environmental consequences of the rapid expansion of the space industry are an active area of research across multiple fields, including astronomy, aerospace engineering, economics, environmental science, law, and policy studies. There are broadly three areas of focus. Astronomy and environmental science studies have evaluated the effects of increased and more-commercialized orbit use on astronomy, dark skies, and cultural heritage (1, 4, 5). Studies in environmental science and aerospace engineering have explored the capacity of orbital space for satellites and constellation architectures, along with the effects of continued orbit use on space debris growth, collision risk, and risks to humans from falling objects (6–9). They also investigate interactions between rocket launches, falling debris, and Earth’s atmosphere (10–13). Law, policy, and economics studies have focused on the legal mechanisms available to address risks on orbit and to people on Earth, the role of economic incentives in space debris creation and collision risk growth, and on developing and quantifying the benefits of policy mechanisms to address space debris and collision risk (14–23).

**Fig. 1.**
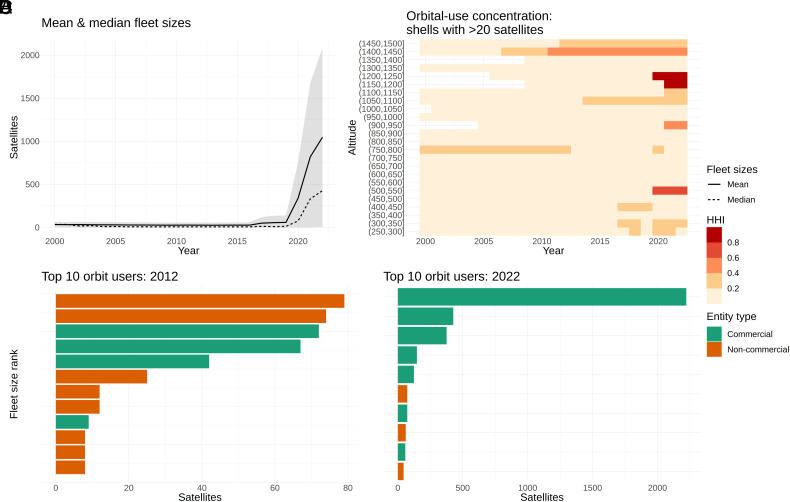
State of orbit use over 2000–2022. (*A*) Growth of average (dotted) and median (solid) satellite fleet sizes across operators (active satellites only). Shaded areas show the average fleet size ±1 standard deviation. (*B*) Herfindahl–Hirschman index (HHI) for active satellite ownership in 50-km orbital shells. The HHI is a standard economic measure of concentration used in competitive and antitrust analysis, computed as the squared share of objects in a shell owned by a single entity (38). Higher numbers indicate that a larger share is owned by a smaller number of actors. Shells with fewer than 20 satellites (10th percentile) are truncated to zero for visualization. (*C*) Top ten operators by active satellite count in 2012. “Noncommercial” entities include civil government, military, and amateur operators. (*D*) Top ten operators by active satellite count in 2022. Data compiled from refs. 2 and 3.

Much less attention has been devoted to the economic consequences for consumers of orbital-use concentration among a few large operators. Yet orbital space is increasingly dominated by a handful of commercial operators who face limited competition. Fig. 1 illustrates this situation: Panel *A* shows the recent growth in fleet sizes, Panel *B* highlights regions of orbital space where a few operators own many satellites, and Panels *C* and *D* show orbital space has gone from being dominated by government-operated satellites to commercially operated satellites. Concentrated use of orbital space may raise issues distinct from “open-access” use, where many small operators ignore their effects on each other. While economic theory and empirical evidence show market concentration can lead to higher prices, lower product quality, and delayed innovation (24–26), it may also improve environmental quality relative to open-access resource use (27–30). How will competition among a small number of constellation operators impact orbital space allocations and service quality? How large are the gains from optimal constellation regulation, and how do they vary with the magnitude of environmental externalities? The growing role of commercial motives in the expansion of the space sector has highlighted a need for coupled-systems frameworks that link physical and economic models of orbit use to answer these questions (31).

To address these knowledge gaps, we build a tractable coupled physicoeconomic model combining microeconomically grounded operator behavior and heterogeneous consumer demand with rich physical structure. We use this model to quantify the economic welfare loss and distortion in orbital allocations from duopoly constellation operators competing for orbital space and market share relative to public utility constellation systems optimally regulated to maximize global public welfare. These public utility systems use orbital space in the public interest, balancing the benefits of better telecommunications service against the costs of externalities like orbital congestion and environmental damages on Earth. This is a classic problem of regulating an oligopolistic sector with environmental externalities (28, 32–34). While it is challenging to address multiple interacting market failures, we find that public utility constellations can increase annual economic welfare from LEO satellite constellations by up to 12%.

Our model is built from a combination of physical and economic first principles as well as reduced-form models that have been validated in other settings (7, 35). We calibrate the model using publicly available data on telecommunications service offered by Starlink and announced by OneWeb (3, 36, 37) as well as economic research on individuals’ willingness-to-pay (WTP) for different features of telecommunications service. We assume that constellations utilize “slotting” architectures to avoid collisions with other orbiting objects but that those avoidance maneuvers disrupt service (7, 8). Our model assumes that megaconstellation operators deploy their systems sequentially and set service prices to maximize their own profits. Economic welfare, or “total surplus,” is measured as the annual sum of economic benefits received by consumers given their WTP for satellite telecommunications service and service prices (i.e., “consumer surplus”) and the profits earned by operators (i.e., “producer surplus”). For example, a total surplus of $10 billion per year means that the difference between the maximum amount consumers were willing to pay and the cost of producing the service was $10 billion.

Though our model is deliberately simplified for tractability and our results are order-of-magnitude estimates, the framework we develop is general and our findings are applicable to existing and planned megaconstellations. Our model lays the foundations for more detailed models of orbit use by megaconstellations. Our results yield insights into how the concentration of orbital space among a small number of commercial players may affect economic and environmental outcomes and supports evidence-based policymaking to promote the sustainable development of the space sector.

## Model

Orbital space allocations are represented by the altitude of the constellation (location) and the number of satellites deployed there (size). Under duopoly use of orbital space, two firms—a first mover (“leader”) and a second mover (“follower”)—provide telecommunications services to consumers on Earth. We consider only two firms for tractability and to represent the near-term situation in orbit. The demand for satellite telecommunications services from each firm is driven by consumer preferences for service quality, which is defined as an index of service availability (more is better), latency (less is better), and bandwidth (more is better). Availability is limited by terrestrial coverage and orbital congestion: The less area the constellation covers at any instant or the more time it spends maneuvering to avoid collisions, the less available it is for consumers. We describe the key model components here and provide details on assumptions, functional forms, and calibration in *SI Appendix*.

### Quality, Availability, Latency, and Bandwidth.

Consumers evaluate telecommunications services based on availability, latency, and bandwidth, which together determine overall service quality. Availability, influenced by coverage area and satellite maneuvers, refers to the fraction of time a service is accessible, with higher availability being more desirable. Coverage area depends on factors such as satellite altitude and beam angle—all else equal, higher altitudes offer greater coverage—while satellite maneuvers are necessary to avoid collisions. Latency is the average time for a signal to travel between the consumer and the satellite, with lower latency preferred. Bandwidth represents the data transmission rate or throughput, with higher bandwidth desired for faster data transfer. Bandwidth is approximately proportional to the ratio of satellites to consumers.

### Location, Size, and Congestion.

We discretize orbital space into a series of nonoverlapping spherical shells. Operators choose a single shell in which to place their constellation. A constellation’s location and size in orbit determine the characteristics of its telecommunications service (i.e., availability, latency, and bandwidth). Lower altitudes allow lower latency but require more satellites to provide full coverage. Larger sizes enable more bandwidth and coverage but increase the number of maneuvers required to avoid collisions and thus reduce availability. We refer to these maneuvers as “orbital congestion.” Lower orbital shells have smaller volumes; all else equal, a constellation at lower altitudes will face greater congestion than it would at higher altitudes. The fundamental tradeoffs faced by satellite constellation operators involve choosing system location and size to optimize service quality. If a system is set too high or is too large, the rise in latency or collision avoidance maneuvers may offset improvements in coverage or bandwidth. On the other hand, a system that is too low or too small may suffer from the reverse.

Following prior works, we use kinetic gas theory to predict close approaches between satellites within an orbital shell (7) (*SI Appendix*). A maneuver is conducted when satellites approach within a specified safety margin of each other. All else equal, higher maneuver safety margins lead to more maneuvers and greater reductions in availability. The maneuver safety margin reflects a combination of technical, behavioral, and regulatory factors, such as constellation slotting architectures (6), positional uncertainty in object trajectories (8), the operators’ risk tolerance, and implementation of avoidance guidelines (39). We assume that all objects use a common safety margin and calibrate it to match open-source analysis of Starlink maneuvers (40). We assume only one satellite involved in a close approach within the safety margin maneuvers, each taking turns.

### Satellites.

Satellites are costly to produce and place in orbit. The cost of a satellite in orbit reflects the cost of materials, energy, and infrastructure required to launch it, maintain the desired altitude, and provide service. The interaction between atmospheric drag—stronger at lower altitudes—and lift energy—more required for higher altitudes—make the cost of a satellite first decline and then increase with altitude (41). The satellite cost function is calibrated to reflect prior literature and public statements regarding Starlink satellites (42, 43). The coverage and bandwidth per satellite at a given altitude are derived from physical first principles and calibrated based on prior literature and analysis of Starlink and OneWeb satellites (36, 43) (*SI Appendix*).

### Competition.

Firms deploy constellations knowing that their location and size choices are irreversible but that their service prices can be adjusted continuously. The irreversibility of location and size choices reflects the high cost of redesigning and relicensing a constellation to operate in a different configuration once it is fully deployed (44, 45). Firms therefore compete in two stages: first in a sequential-move location-and-size-choice game and second in a simultaneous-move price-setting game. The leader anticipates the follower’s entry and chooses their location and size to maximize their own profits, while the follower chooses their location and size to maximize their own profits given the leader’s choices. The leader thus alters the follower’s location and size choice to their own advantage. Firms are forward-looking and anticipate the outcomes of the pricing subgame when choosing locations and sizes.

### Consumers.

Consumers choose the service that provides them with the most satisfaction given service characteristics and prices (i.e., maximizes their utility). Consumers are heterogeneous and value service quality differently. We calibrate their preferences to reflect recent consumer survey results (46). We focus on end consumers rather than intermediaries. The market share captured by each firm is determined by the consumer who is indifferent between service offerings. Consumers who place a higher value on service quality choose the firm with higher service quality. All else equal, the more consumers a system serves, the less bandwidth is available to all consumers (i.e., there are network congestion effects). To reflect a near-term scenario with two operational constellations, in the benchmark case, we consider a market with 10 million consumers globally.

### Scenarios.

We consider three scenarios: the duopoly equilibrium, in which firms choose locations, sizes, and prices to maximize their profits and consumers choose a service to maximize their utility; and two types of public utility systems, with one or two constellations. We use the term “public utility system” to refer to a system of constellations, in a given scenario, which are designed and regulated to maximize global economic welfare. A one-constellation public utility system shows the potential gains from regulation while providing equitable access to all consumers, while a two-constellation public utility system shows the potential gains from regulation while providing differentiated service to consumers. The optimal public utility system is the one which provides greater economic welfare. We use a public utility framework to consider optimal use of orbital space since, due to high fixed costs, perfect competition in this market is unlikely. We use these scenarios to quantify the maximum benefits of regulating orbit use relative to the status quo. In all scenarios, we include the background traffic of objects (excluding Starlink and OneWeb) recorded by Space-Track.org as of December 26, 2022 (2). We assume that there are no environmental damages on Earth from satellite constellations in the benchmark calibrations and conduct sensitivity analysis over the magnitude of these damages to identify key thresholds. The public utility systems internalize the environmental costs of constellations when designing the systems.

## Results

Table 1 shows the constellation design parameters under each scenario in the benchmark calibration with no environmental damages (*SI Appendix*). In the duopoly equilibrium, the leader anticipates the follower’s entry and launches a larger constellation at a lower altitude (29,750 satellites at 500 km), forcing the follower to choose a smaller constellation at a higher altitude (1,945 satellites at 603 km). These design choices give the leader higher availability and bandwidth and lower latency than the follower, enabling the leader to capture the majority of the market—particularly the most lucrative segment. Both the duopoly leader and the one-constellation public utility system are placed near the cost-minimizing altitude of 500 km (*SI Appendix*). By moving first, the duopoly leader is able to claim the better location. The spacing in the duopoly case reflects the logic of competition in vertically differentiated markets: Increasing the differentiation between the service offerings increases both firms’ profits, as it decreases the “toughness” of competition between the two for indifferent consumers (*SI Appendix*).

**Table 1.** Comparisons of constellation designs under different scenarios in the benchmark calibration

Scenario	Duopoly		Two public utility constellations		
Constellation	Leader	Follower	Larger	Smaller	One public utility constellation
Mean altitude [km]	500	603	480	515	495
Size [sats]	29,750	1,945	26,365	18,199	45,151
Latency [ms]	33	34	33	33	33
Bandwidth [Mb/s]	90	43	122	97	111
Availability [%]	100	69	100	100	100
Market share [%]	83	17	53	47	100

All values are rounded to the nearest integer.

In the two-constellation public utility system, constellations are placed at lower altitudes (480 and 515 km) and differently sized compared to the duopoly system—the larger system is smaller than the duopoly leader’s (26,365 satellites), while the smaller system is larger than the duopoly follower’s (18,199 satellites). The one-constellation public utility system is the largest of all (45,151 satellites) and placed slightly lower than the duopoly leader’s (495 km). The two public utility constellations are located around the cost-minimizing orbital altitude (500 km) with just enough separation distance to avoid between-constellation congestion. As both one- and two-constellation public utility systems use similar altitudes, the marginal cost of an additional satellite under both systems is comparable, leading to similar total system sizes. Despite having fewer satellites, market segmentation and reduced network congestion allow the larger constellation in the two-constellation system to offer higher bandwidth to consumers who value service quality more. While this benefits the larger constellation’s users, it comes at the expense of lower bandwidth for users of the smaller constellation, who value service quality less. Both public utility systems deliver availability and latency comparable to the duopoly leader’s.

**Fig. 2.**
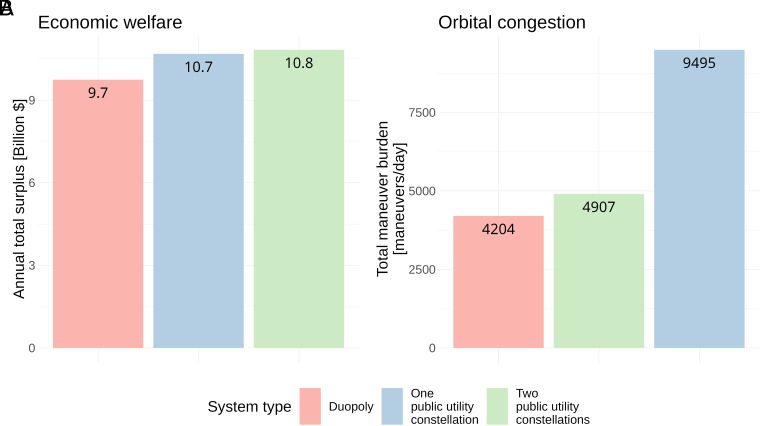
Equilibrium and public utility constellation economic welfare and congestion under benchmark calibration. (*A*) Annual economic welfare generated by each system type. (*B*) Daily expected congestion for constellations under each scenario.

Fig. 2 shows the aggregate annual global economic welfare (Panel *A*) and orbital congestion (Panel *B*) created by each system type in the benchmark calibration. Welfare is computed as the global annual total surplus generated by the system as a whole. Orbital congestion (i.e., collision avoidance maneuvers within and between constellations) is computed as the expected daily number of maneuvers for each system (*SI Appendix*). We project that shifting from duopoly constellations to a one-constellation public utility system would increase annual economic welfare by around $1 billion USD (roughly 10%), while a two-constellation public utility system would increase annual global economic welfare by around $1.1 billion USD (roughly 12%). Though the larger public utility constellation sizes and lower locations increase welfare, they also increase orbital congestion. Compared to duopoly constellations, we project that a one-constellation public utility system would induce around 5,291 additional collision avoidance maneuvers per day (roughly a 126% increase), while a two-constellation public utility system only induces around 703 additional maneuvers per day (roughly a 17% increase). These maneuvers are conducted to avoid collisions between satellites in the same constellation—both in equilibrium and under public utility designs the constellations are placed far enough apart that no maneuvers are necessary to avoid collisions between satellites from different constellations. In the case of the two-constellation public utility system, the spacing is just sufficient given the discretization of orbital space to avoid between-constellation congestion (*SI Appendix*).

**Fig. 3.**
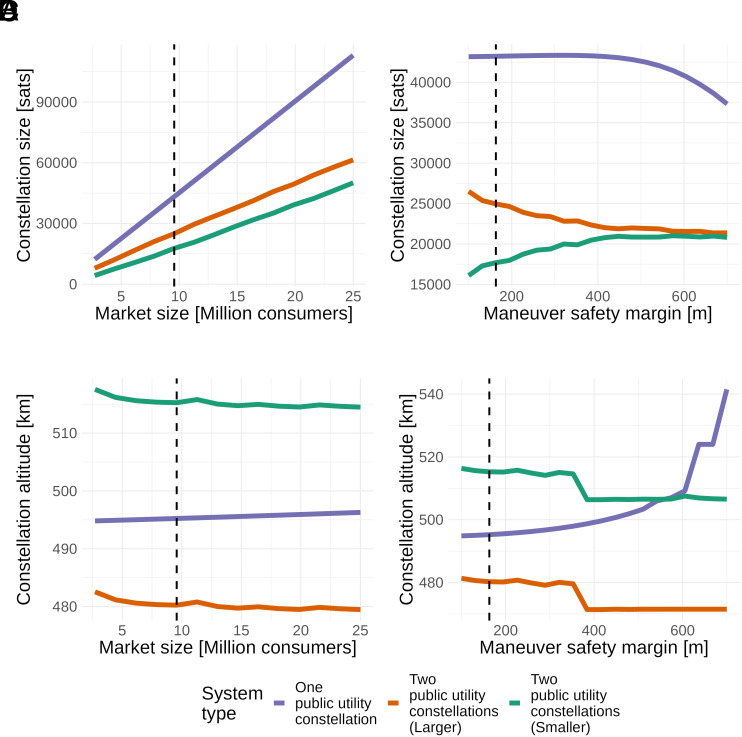
Public utility constellation sizes and locations as number of consumers served and safety margin increase. (*A* and *B*) show the constellation size changes related to the number of consumers served and safety margin increase. (*C* and *D*) show the location changes related to the number of consumers served and safety margin increase. The dashed vertical lines show the benchmark calibration. Irregularities in the two-constellation problem’s optimization surface introduce numerical artifacts in the solution paths.

Fig. 3 shows how the sizes and locations of public utility constellations change as the number of consumers served (Panels *A* and *C*) and maneuver safety margin (Panels *B* and *D*) increase. At very low market sizes, a single public utility constellation can serve the market most efficiently, and the one- and two-constellation systems are identical (Panels *A* and *C*). As the market grows, larger constellations are needed to provide sufficient service quality (Panel *A*). Despite the increase in orbital congestion, the public utility systems only raise altitude slowly as market size increases (Panel *C*). In the two-constellation public utility system, the constellations are separated by the minimal distance necessary to avoid between-constellation congestion (Panel *C*). When the safety margin is low, the one-constellation public utility system can be larger (Panel *B*) and placed at lower altitudes (Panel *D*), providing lower latency and higher bandwidth with minimal congestion. As the safety margin increases, it becomes necessary to make it smaller to reduce orbital congestion. The two-constellation public utility system does not follow this pattern. As the safety margin increases, satellites are reallocated from the larger constellation to the smaller one (Panel *B*), reducing overall orbital congestion with smaller impacts on service quality. Both constellations are moved lower, reducing latency and partially offsetting the effect of lower availability (Panel *D*).

**Fig. 4.**
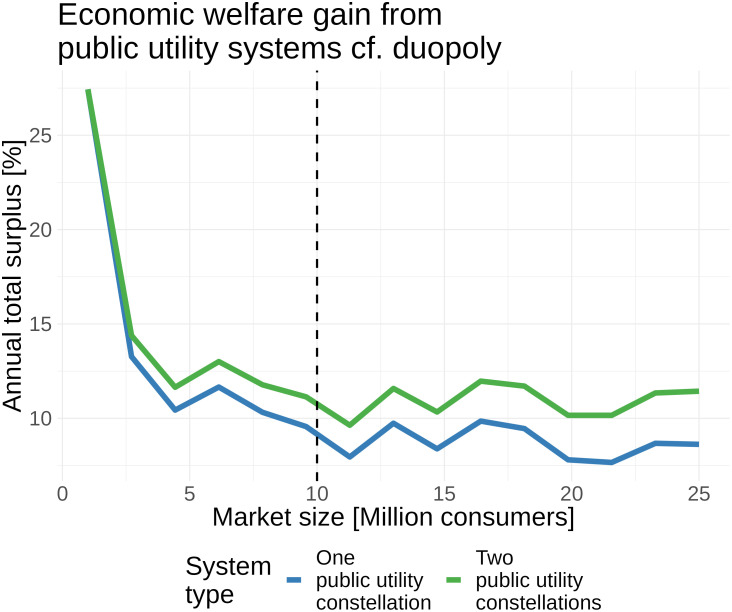
Percentage gain in economic welfare from public utility systems relative to the duopoly equilibrium. The orbital shell discretization for the duopoly problem introduces numerical artifacts. The dashed vertical line shows the benchmark calibration.

Fig. 4 shows how the economic welfare gain from public utility systems (relative to duopoly) scales with the number of consumers served. The two-constellation public utility system is economically optimal, providing uniformly greater welfare up to 25 million consumers served (the maximum we simulate). The percentage gain from the two-constellation system is decreasing in the number of consumers served—from around 25% at 1 million consumers to around 10% at 25 million consumers. The percentage gain from the two-constellation system decreases to a little over 10% when 25 million consumers are served globally. The percentage gain from a one-constellation system follows the trend of the two-constellation system, reaching a little over 5% with 25 million consumers. The scaling with the number of consumers served reflects the fact that total welfare is increasing in the market size under all system types including duopoly. Thus, the monetary value of a 10% gain at 20 million consumers exceeds the value of a 20% gain at 10 million consumers (*SI Appendix*).

**Fig. 5.**
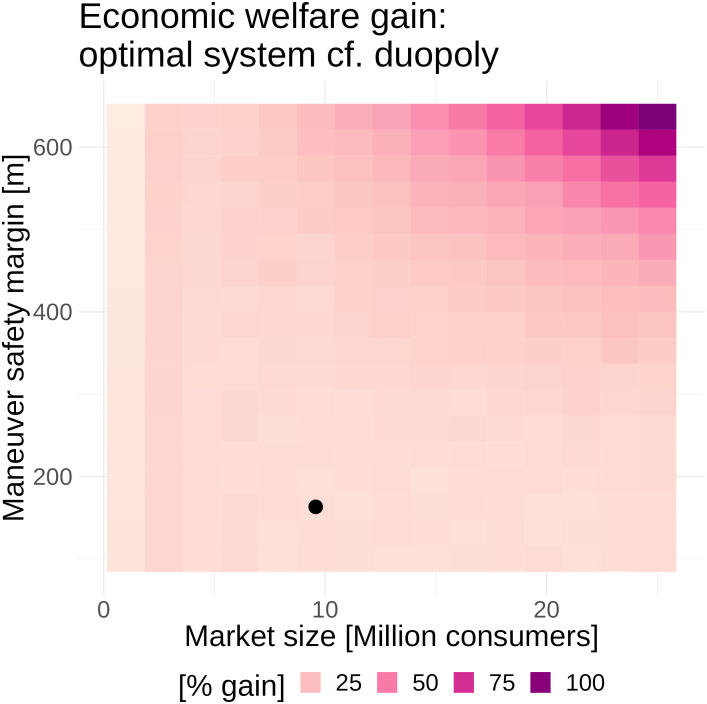
Percentage gain in economic welfare from the economically optimal two-constellation public utility system relative to the duopoly equilibrium. The black dot shows the benchmark calibration.

Fig. 5 explores how the gains from the economically optimal two-constellation system scale with both market size and maneuver safety margin. Since we assume all avoidance maneuvers are successful, reduced safety margins correspond to a best-case for better avoidance technologies/practices (i.e., improvement at zero cost). These include better space situational awareness, slotting architectures, control systems, and satellite coordination. Both the percentage and absolute monetary gains are greatest at large market sizes and large safety margins.

**Fig. 6.**
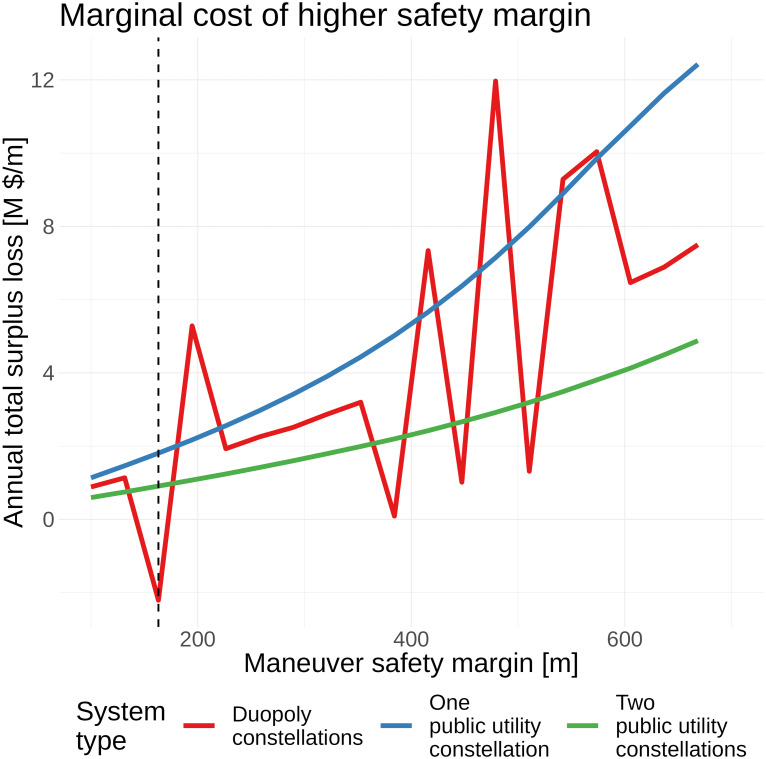
Reduction in total welfare per unit increase in maneuver safety margin. Numerical artifacts are introduced in the duopoly calculation due to orbital shell discretization. The dashed vertical line shows the benchmark calibration.

Fig. 6 shows the marginal welfare cost of higher safety margins, i.e., the change in economic welfare from increasing the safety margin. Higher safety margins imply greater orbital congestion, as the same distribution of orbiting objects requires more maneuvers. The marginal welfare cost is generally increasing in the safety margin, though it is highest for the one-constellation public utility system. This is driven by the fact that the one-constellation system does not spread satellites across multiple locations. As the safety margin increases, it is forced to reduce the total number of satellites faster to manage congestion (Fig. 3*B*). The marginal welfare cost of higher safety margins is lowest under the two-constellation public utility, since it efficiently reallocates satellites across constellations to maintain service quality. The duopoly faces a marginal welfare cost between the two public utility designs, as it disperses satellites across multiple orbits but does so inefficiently.

**Fig. 7.**
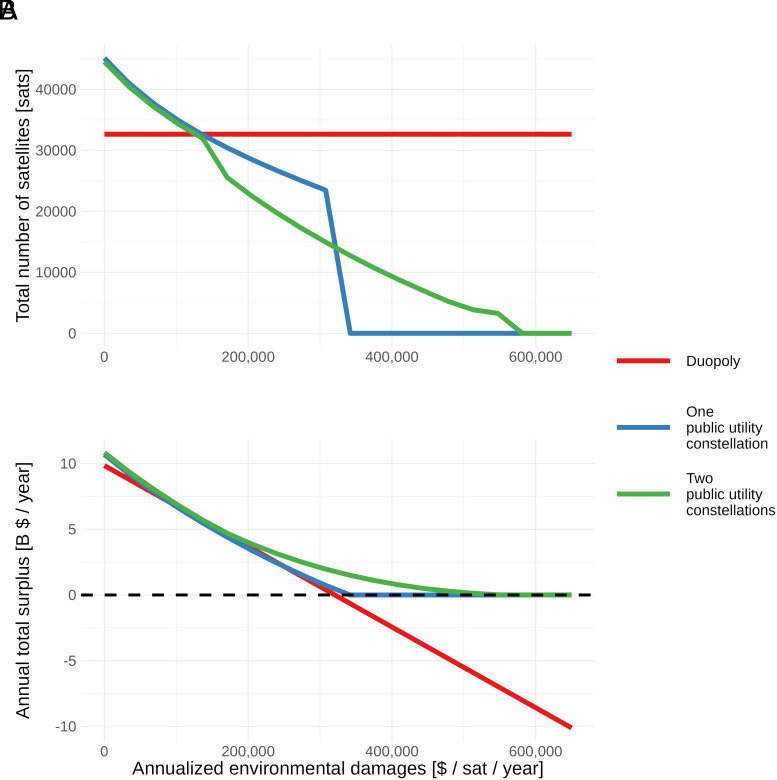
Effects of greater environmental damages on optimal constellation designs and economic welfare.

Finally, Fig. 7 illustrates how environmental damages affect public utility and duopoly constellation sizes (Panel *A*), as well as their subsequent effects on economic welfare (Panel *B*). These damages encapsulate the annualized value of various environmental externalities other than orbital congestion, such as rocket emissions, orbital debris, ozone layer degradation, and reentry impacts on people and property (*SI Appendix*).

Panel A shows that the public utilities progressively decrease their system sizes as environmental damages increase, with total numbers of satellites matching the duopoly at approximately $150,000 in damages. The two-constellation public utility then transitions to a single constellation and no longer provides full-market coverage. This causes the total number of satellites in the (formerly) two-constellation public utility to dip below that of the one-constellation public utility. The two-constellation system continues until the damages reach around $600,000, at which point even a single satellite generates negative economic welfare. In contrast, the one-constellation public utility maintains its market-wide nondifferentiated service until damages reach around $330,000. Beyond this point, the minimal constellation necessary to provide desirable service to the whole market generates negative welfare. Since the one-constellation public utility must provide uniform service to the entire market or none at all, it ceases operations. The system altitudes change little or not at all (*SI Appendix*).

Panel B shows the net economic welfare generated by the economically optimal two-constellation system and the duopoly as environmental damages increase. At around the same level of damages where the one-constellation public utility shuts down, the duopoly’s use of orbital space generates net negative economic welfare. In contrast, the two-constellation public utility continues to provide positive economic welfare until it shuts down, reducing its size to avoid generating more environmental damages than surplus from telecommunications service. There is also a range of annualized damages—roughly $100,000 to $250,000—where the duopoly provides greater welfare than the one-constellation public utility. This is due to the gains from market segmentation (even under the duopoly) relative to providing uniform service quality to all consumers.

## Discussion

Space in low-Earth orbit is increasingly concentrated in the hands of a small number of competing commercial telecommunications firms. Our analysis suggests that this competition will be a critical factor determining how orbital space is allocated and that improving economic welfare from LEO telecommunications services will require altering the allocation of orbital space. Failing to do so will limit the degree to which economies benefit from the use of low-Earth orbit.

Fundamentally, there are two interacting market failures in orbit use. The first, identified in prior literature, is the open-access problem. Lacking exclusive property rights to orbital slots, operators do not account for how their behaviors impact other orbit users (15, 20, 21). On its own, this market failure can be remedied through environmental policy, e.g., externality-correcting taxes on orbiting satellites. The second, which we address here, is oligopolistic competition between orbit users. Oligopolistic megaconstellation operators utilize orbital space to exercise market power. On its own, this market failure can be remedied through competitive or antitrust policy, e.g., public utility regulation of megaconstellations. These market failures cut in opposite directions: While the open-access problem will lead to an excessive number of objects in orbit, oligopolistic competition will lead to too few satellites in orbit.

The large spacing and size differences between the duopoly constellations reflect the firms’ incentives to increase product differentiation and reduce the toughness of price competition (35, 44). The low service quality levels in the duopoly equilibrium reflect firms’ incentives to minimize production costs. The combined effect of these incentives is lower service quality—a common feature of oligopolistic competition in markets for differentiated products (35, 44, 47, 48)—and lower orbital congestion. Such “excessive conservationism” is a common feature of oligopolistic use of natural resources (27, 29, 30, 49). Regulating natural resource use under multiple market failures is challenging, often requiring more-complex policies than if the market failures existed in isolation (28, 32–34).

We have focused on oligopolistic competition with two constellation operators, reflecting the near-term situation with Starlink and OneWeb. Public filings suggest that more firms are likely to enter the market soon, e.g., Amazon’s Kuiper and Telesat’s Lightspeed (37). Our analysis provides a useful reference point for understanding how competition between these operators is likely to evolve. The economic literature suggests that entry into such markets may be limited (partly due to choices made by first-movers and incumbents) and that more entry is not necessarily welfare-enhancing (50, 51).

Though we abstract from the fixed costs of deploying a constellation—e.g., designing the system, obtaining regulatory clearances for ground stations and spectrum licenses—to focus on competition between two firms, the fixed costs can be substantial. These fixed costs make perfect competition—with many constellation operators serving small portions of the market, none able to individually affect market outcomes—unlikely to materialize. Indeed, it is an open question whether the market can support multiple satellite constellations even if they target different market segments. History suggests that large LEO satellite constellations tend to face significant economic challenges—Iridium, an early example, went bankrupt in the early 2000s (52). More recently, OneWeb filed for bankruptcy in 2020 (53). These considerations suggest that imperfect competition is likely an important feature to consider when studying orbit use.

Finally, we show that the environmental damages caused by satellites over their lifecycle play a critical role in determining the relationship between oligopolistic and economically optimal orbit use. When environmental damages are low, imperfect competition causes too few satellites to be in orbit relative to the optimal public utility system. As damages increase, the economically optimal system shrinks while the duopoly fleet remains unchanged, resulting in too many satellites in orbit relative to the optimal public utility system.

### Limitations and Future Research.

Following prior work on constellation slotting architectures, we assume that all collision avoidance maneuvers are successful (7). While this assumption has been empirically validated thus far, eventually some are likely to fail and generate debris fragments which induce further maneuvers. Such outcomes will likely reduce the sizes of the duopoly and public utility systems and may create a further incentive to place constellations at lower altitudes, where debris will decay and burn up in Earth’s atmosphere more rapidly. We also do not account for congestion created by the process of replenishing constellations (e.g., orbit-raising maneuvers) or the congestion created by avoiding satellites which are deorbiting. These issues will multiply as more constellations are deployed and the demand for satellite telecommunications services grows. Incorporating such issues will again likely alter the design of the economically optimal public utility constellation system. Future research in this area should incorporate debris and collision risk dynamics into models of strategic orbit use behaviors. Finally, we assume that consumers and bandwidth demands are uniformly distributed on the globe and over the day. Spatial and temporal nonuniformities, e.g., reduced service usage at night boosting peak bandwidth, may create further opportunities for market segmentation (*SI Appendix*).

Though we do not explicitly model debris formation and decay, the public utility constellation designs we calculate are at relatively low altitudes. Such low placement should ensure that orbital debris produced burns up in the atmosphere within 25 years, consistent with current international disposal guidelines (54). However, using the atmosphere to dispose of satellites comes at the cost of depositing large quantities of satellite materials in the upper atmosphere, damage to the ozone layer, and reentry risks for people and property on Earth (10–13). Large satellite constellations also impose costs on ground-based observation systems (1, 4), i.e., light pollution. Though we identify an aggregate cutoff level of annualized damages such that the optimal public utility fleets are smaller than the duopoly fleet ($150,000 per satellite per year), our model does not speak to how these damages will be distributed. Unfortunately, detailed estimates of the environmental damages and distributions of these externalities are not yet available. While best practices for life cycle assessment of large satellite constellations are still developing, existing research and guidelines note that factors such as the propellant and motor used in rockets deploying the constellation can strongly affect the system’s environmental damages (55, 56). Future research should study these damages and incorporate them into more detailed physicoeconomic models of orbit use.

Despite these issues, satellite constellations may help spur innovations in small satellite designs, which can have positive effects for scientific and astronomical discovery (5). On the other hand, long-standing economic literature has identified oligopolistic use of natural resources as a barrier to technological innovation (24, 49). Developing better understanding of policy designs to address interactions between multiple market failures—particularly environmental externalities, imperfect competition, and positive innovation spillovers—may prove useful for future sustainable growth policies in other settings.

We have also abstracted from issues of national strategic uses of orbital space. While satellites owned and operated by national militaries are a declining share of satellites in orbit (largely due to the growth of megaconstellations), militaries and governments also act as important customers for megaconstellation operators, and megaconstellations may serve important national strategic interests (57). Governments facing such incentives may prefer constellation operators they purchase from to not serve other governments, or even to have their own systems, driving demand for multiple systems. Such demand is evident in discussions around a European constellation, the UK government’s interest in supporting the purchase of OneWeb following their bankruptcy in 2020 [the UK government now holds a roughly 19% stake in OneWeb (58)], and potentially in the Chinese government’s support for the GuoWang system (59–61). While our findings regarding economic welfare for civilian consumers are robust to such use cases, they raise the important point that governments may be willing to trade economic welfare for other objectives when using orbital space.

Finally, we use the term “public utility system” to refer to a system of constellations (one or two, depending on the scenario) which are designed and regulated to maximize global economic welfare. While public utility-like uses of space resources have been considered for positioning, navigation, and timing services or Earth observation data, we are unaware of similar proposals for telecommunications megaconstellations (62, 63). We abstract from regulatory issues analyzed in the economic literature, such as asymmetric or incomplete information, capital bias, regulatory capture, and management of network effects (64–69). For example, SpaceX is currently one of the only firms with reusable rockets and is vertically integrated with a satellite constellation. Similarly, Amazon’s planned satellite constellation would be vertically integrated with several internet services such as entertainment, shopping, and cloud computing. Such integration may provide different incentives for innovation between the private and the public sectors. Given the unique environmental, economic, and geopolitical features of orbit use, optimal public utility regulation of satellite megaconstellations may look very different from public utility regulations in other sectors—even from terrestrial telecommunications providers. Indeed, economic theory suggests market power should be regulated on a case-by-case basis (the “rule of reason” approach) rather than through rigid “per se” rules across industries (70).

It may be possible to conduct such regulation under existing space governance institutions. In particular, Article VI of the Outer Space Treaty requires signatories to authorize and continually supervise the activities of their space industries (71). This suggests the potential for nationally administered but internationally coordinated constellation regulatory systems. Such coordination—extending to radio spectrum allocations, space traffic management, and orbital debris mitigation and remediation—poses significant challenges, particularly given its impacts on the distribution of service access and its national strategic implications. Future research should study international satellite constellation regulatory competition and seek strategies for enhancing its outcomes.

Oligopolistic competition between orbit users will drive inefficient orbital-use patterns, with low and highly unequal service quality. These inefficiencies persist even with improvements in collision avoidance technologies and practices. Environmental externalities like rocket emissions, debris accumulation and reentry, and light pollution worsen the inefficiency of oligopolistic orbit use. Public utility regulation of constellations could substantially improve global economic welfare from orbit use. These benefits grow as orbital space grows more congested and constellations serve larger markets. While there is much to be done to design and implement these regulations, recognizing and quantifying the tradeoffs and complementarities involved is an important step forward.

## Supplementary Material

Appendix 01 (PDF)

## Data Availability

Code and simulation results’ data (72), as well as code and empirical data used to generate Fig. 1 (73), have been deposited in Middlebury Institutional Repository.
