# Supplementary material for: Oligopoly competition between satellite constellations will reduce economic welfare from orbit use
Source: Proc Natl Acad Sci U S A. 2023 Oct 16;120(43):e2221343120. doi: 10.1073/pnas.2221343120 (PMC10614827; doi:10.1073/pnas.2221343120)
Supplement: Supplementary file 1 — Appendix 01 (PDF) [file pnas.2221343120.sapp.pdf]

Supporting Information for "Oligopoly competition between satellite constellations will reduce economic welfare from orbit use"

July 24, 2023

Contents

1 Model overview **2**

2 Model components and calibration **2**

2.1 Consumer demand and firm pricing . . . 3

2.2 Constellation service quality and cost . . . 4

2.2.1 Service quality . . . 5

2.2.2 Earth Coverage . . . 6

2.2.3 Latency . . . 7

2.2.4 Bandwidth . . . 8

2.2.5 Satellite unit costs . . . 8

2.3 Avoidance maneuvers and service availability . . . 9

2.4 Solution concepts and algorithms . . . 11

2.4.1 Duopoly equilibrium . . . 11

2.4.2 Social optimum . . . 12

2.4.3 Algorithms . . . 13

2.4.4 Orbital shell discretization . . . 14

2.5 Parameter values . . . 15

3 Sensitivity analysis **17**

3.1 Market size and maneuver safety margin . . . 17

3.2 Preference parameters . . . 18

3.3 Environmental damages . . . 19

1 Model overview

We model competition between firms for orbital space and telecommunications market share as a two-stage game. Constellation operators are indexed by $i = F, L$. In the first stage, they compete in a sequential-move (Stackelberg) game to choose their constellations' altitude and size. Firm L (the Leader) deploys their constellation first and firm F (the Follower) deploys second.[1] Both players are rational, so the Follower best-responds to the Leader's choice and the Leader anticipates the Follower's best-response when designing their constellation. With their constellations deployed, in the second stage, firms play a simultaneous-move price-setting game to compete for demand from a mass of N consumers.[2]

Deploying a constellation means choosing a number of satellites Q_i to maintain and a mean orbital altitude h_i to place them. These design choices determine the overall service quality x_i. Service quality is a function of the constellation's global Earth coverage α_i, congestion loss β_i, latency L_i, and bandwidth S_i. Earth coverage is increasing in altitude and constellation size. Congestion loss is increasing in the constellations' sizes and proximity to each other. Latency is increasing in altitude. Bandwidth is increasing in constellation size.

The annualized unit cost of manufacturing, deploying and maintaining a satellite at altitude h_i, inclusive of ground stations and other support infrastructure, is $C(h_i)$. We assume it is first decreasing (for $h_i < \underline{h}$) and then increasing in altitude (for $h_i < \underline{h}$). This is explained by the countervailing facts that a higher altitude requires more lift energy at launch, but a lower altitude requires more fuel during operational lifetime to offset drag [De Pater and Lissauer, 2015]. [3] The annualized total cost of a constellation is $C(h_i)Q_i$.

Consumers purchase one unit of telecommunications service and are characterized by their preference for quality, θ. A type θ consumer has utility $u(\theta, i) = \theta x_i - p_i$ from purchasing satellite service from operator i, given service quality x_i and price p_i. We assume θ is uniformly distributed between $\underline{\theta}$ and $1 + \underline{\theta}$ in the population.

In the following sections, we construct the coupled physico-economic model from a combination of physical and economic first principles and empirical data.

2 Model components and calibration

In this section we describe the components of the model, their calibration, and the solution concepts and algorithms in more detail.

[1] This could be the case if, for example, the Leader has earlier access to a cost-reducing technology which eventually gets imitated. See [Prescott and Visscher, 1977] for more on the realism of sequential- vs simultaneous-move games in economics.

[2] We assume that the target population is consumers living in areas with low population density, who do not have access to the Internet by land-based means.

[3] The annualized unit cost of a satellite may well differ between the two competitors, either because of different design choices or because of different launch costs. We neglect these aspects below, due to the lack of precise data.

2.1 Consumer demand and firm pricing

Consumers choose between purchasing one unit of service from the Leader or the Follower. The mass of N consumers is uniformly distributed in preference for satellite service quality, θ, over $[\underline{\theta}, 1+\underline{\theta}]$. We assume that $\underline{\theta}$ is large enough, so that all targeted consumers will be willing to purchase satellite service.

Assume that, in equilibrium, the Leader offers a better quality than the Follower ($x_L > x_F$). We will show that the firm with the higher quality charges a higher price ($p_L > p_F$).[4] The consumer who is indifferent between the two services has preference parameter θ^* such that

$$\theta^* x_L - p_L = \theta^* x_F - p_F, \tag{1}$$

which can be solved to yield

$$\theta^* = \frac{p_L - p_F}{x_L - x_F}. \tag{2}$$

If $\underline{\theta} < \theta^* < 1+\underline{\theta}$, the demands for each service are

$$D_L = \left(1 - \frac{p_L - p_F}{x_L - x_F}\right) N, \tag{3}$$

$$D_F = \frac{p_L - p_F}{x_L - x_F} N. \tag{4}$$

The firms set prices simultaneously to maximize their individual profits,

$$\max_{p_L} \pi_L = p_L \left(1 + \underline{\theta} - \frac{p_L - p_F}{x_L - x_F}\right) N - C(h_L) Q_L, \tag{5}$$

$$\max_{p_F} \pi_F = p_F \left(\frac{p_L - p_F}{x_L - x_F} - \underline{\theta}\right) N - C(h_F) Q_F. \tag{6}$$

Their first-order conditions are

$$\left(1 + \underline{\theta} - \frac{p_L - 2p_F}{x_L - x_F}\right) N = 0, \tag{7}$$

$$\left(\frac{p_L - 2p_F}{x_L - x_F} - \underline{\theta}\right) N = 0, \tag{8}$$

$$\tag{9}$$

yielding (Nash equilibrium) prices

$$p_L = \frac{2+\underline{\theta}}{3}(x_L - x_F), \tag{10}$$

$$p_F = \frac{1-\underline{\theta}}{3}(x_L - x_F), \tag{11}$$

[4] When qualities are identical, the pricing stage reduces to a Bertrand duopoly game between identical firms.

and demands

$$D_L = \frac{2+\underline{\theta}}{3}N, \tag{12}$$

$$D_F = \frac{1-\underline{\theta}}{3}N. \tag{13}$$

The optimized profits are

$$\pi_L = \frac{(2+\underline{\theta})^2}{9}(x_L - x_F)N - C(h_L)Q_L, \tag{14}$$

$$\pi_F = \frac{(1-\underline{\theta})^2}{9}(x_L - x_F)N - C(h_F)Q_F. \tag{15}$$

Notice that the equilibrium prices in equations 10 and 11 and equilibrium profits in equations 14 and 15 are increasing in the degree of quality differentiation between the two firms (i.e. p_i and π_i are increasing in $x_L - x_F$). Thus, constellation operators face incentives to differentiate their service offerings when choosing constellation design parameters which determine quality (altitude and size). This incentive raises the potential that firms over-differentiate in equilibrium.

2.2 Constellation service quality and cost

Service quality depends on the constellations' availability, coverage, latency, and bandwidth. We show below that they are determined by the constellation own altitude and size, but also by the other constellation altitude and size. For tractability, we assume that consumers are uniformly distributed over the Earth. See Osoro and Oughton [2021] for more detailed analysis of how non-uniformities in the geographical distribution of consumers affects the distribution of service quality at different locations. We give some brief intuition below for how these features may matter in a model like ours.

It is important to note that the distribution of consumers is non-uniform in three senses: geographically (population densities vary according to location on the globe), economically (consumer characteristics vary according to location on the globe), and temporally (the same location has different demand characteristics at different times). Since low-Earth orbit (LEO) satellites revolve around the Earth, designing a constellation to only serve specific customers at specific times (e.g. where and when geographic and economic features are favorable) is challenging if not impossible. Still, these non-uniformities may lead to deviations from our modeling results in practice.

On one hand, our model implicitly accounts for some of these non-uniformities. The distribution of consumer preferences (i.e. θ) we use may already both represent economic heterogeneity *within* and *between* regions. For example, to the extent that high-WTP consumers are concentrated in particular areas (such as the global North), our model allows the firms to target those consumers through service offering characteristics. Similarly, to the extent that demand is lower at night, peak throughput (bandwidth per consumer) for subscribers may be higher than during the day, but average or worst-case peak throughput can still be calculated as in equation 25 below. On

the other hand, some non-uniformities may affect characteristics that are integrated over physical space (e.g. bandwidth per consumer) more substantially. For example, a uniform distribution will overstate the bandwidth available in more-densely populated regions and understate bandwidth in more-sparsely populated regions. However, this is unlikely to substantially affect our main conclusions or estimates, given that the estimates are at the annual level and that constellation operators will likely target consumers living in sparsely populated areas not served by terrestrial means—a more homogeneously-distributed group than the consumer population at large.

2.2.1 Service quality

We assume that consumers evaluate telecommunications service from a constellation i along three dimensions: availability, latency and bandwidth. The willingness-to-pay (WTP) x_i of a representative consumer (i.e. with type $\theta = 1$) for a service with availability α_i, latency L_i, and bandwidth S_i will have the form

$$x_i = F(\alpha_i)G(L_i, S_i), \tag{16}$$

where F is a function reflecting the fraction of WTP preserved given partial service availability and G is a function reflecting WTP for fully-available service. We assume that F and G satisfy the following conditions:

1. Consumers will be willing to pay nothing for service which is never available: $F(0) = 0$;
2. Consumers prefer greater availability: $F'_\alpha(\alpha) > 0$ for all $\alpha \in [0,1]$;
3. A representative consumer (i.e. with type $\theta = 1$) will be willing to pay $G(L,S)$ when service with latency L and bandwidth S is always available : $F(1) = 1$;
4. Consumers prefer less latency and higher bandwidth: $G'_L(L,S) < 0$ and $G'_S(L,S) > 0$ for all $L \geq 0$ and $S \geq 0$.

We assume consumers have positive WTP for a service with latency no greater than $\bar{L}$, with bandwidth preference parameter a_S and latency preference parameter a_L. These parameters are calibrated such that a typical consumer's WTP for the service is 1500 $/year. We use the following specifications:

$$F(\alpha) = \alpha^2, \tag{17}$$

$$G(L,S) = a_L(\overline{L} - L)\frac{S^2}{a_S + S^2}, \tag{18}$$

where $\alpha \in [0,1]$, $L \geq 0$, and $S \geq 0$. The specification of F implies that consumers strongly prefer higher availability. The specification of G implies that, ceteris paribus, consumers have constant marginal WTP for lower latency and a diminishing marginal WTP for larger bandwidth. Latency and bandwidth are assumed to contribute multiplicatively to overall WTP. That is, while consumers are willing to substitute between latency and bandwidth to some extent, there are also complementarities between the two. a_S has units of bandwidth squared ((Mb/s)^2), $\bar{L}$ has units of latency (ms), and a_L has units of WTP per unit latency ($/ms).

We calibrate a_L, a_S and $\overline{L}$ to be consistent with the following data:

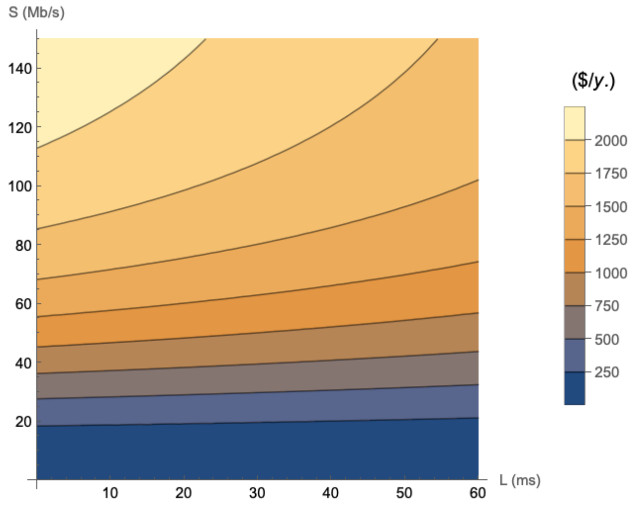

Figure S1: Maximum willingness to pay of a representative consumer ($\theta = 1$). The contour map reflects that greater bandwidth is a good while greater latency is a bad.

(i) A standard internet service provides a latency of 25 ms, a bandwidth of 75 Mb/s, and is available 100% of the time;

(ii) A representative consumer is willing to pay a maximum of 1500 $/y. to subscribe for a standard internet service;

(iii) The willingness to pay for a lower latency is about 6 $/year/ms [Liu et al., 2018];

(iv) The willingness to pay for an increased bandwidth amounts to 168 $/year, from 4 Mb/s to 10 Mb/s, and 288 $/year, from 10 Mb/s to 25 Mb/s [Liu et al., 2018]).

The values of a_L, a_S, and $\bar{L}$ chosen to approximately match these targets are listed in Table S2. Figure S1 gives a graphical representation of the marginal WTP of a representative consumer ($\theta = 1$) for a fully-available ($F \equiv 1$) service supplying latency L (x-axis) and and bandwidth S (y-axis). Later, we will conduct a sensitivity analysis with respect to preference parameters a_L, a_S and $\overline{L}$. In order to obtain relevant and comparable outcomes, we will restrict our attention to sets of parameters satisfying requirements 1, 2 and 3 above.

2.2.2 Earth Coverage

We assume that each satellite of a constellation with average altitude h_i can cover a circle of radius $h_i \tan(\phi/2)$ and area $\pi h_i^2 \tan(\phi/2)^2$, where ϕ is the average angle of the beam from the satellite to the surface. The surface area of the Earth is πR^2, so the minimum number of satellites needed to fully cover the Earth at any instant is approximately

$$\underline{Q}(h_i) = \frac{R^2}{h_i^2 \tan(\phi_i/2)^2}. \tag{19}$$

The constellation consists of Q_i satellites. However, at a given instant, due to avoidance maneuvers, $\beta_i Q_i$ satellites are "turned off" (not providing service) on average (see below for the definition

and calculus of β_i). If the remaining constellation is smaller than the minimum covering number ($(1-\beta_i)Q_i < \underline{Q}(h_i)$), each operational satellite will serve an area of $\pi h_i^2 \tan(\phi/2)^2$ and the system will have gaps in coverage (i.e. areas which are not covered at some point of the day). If the remaining constellation is larger than the minimum covering number ($(1-\beta_i)Q_i \geq \underline{Q}(h_i)$), it will have full coverage. Each operational satellite then covers a circle with radius $R/\sqrt{(1-\beta_i)Q_i}$ and area $\pi R^2/((1-\beta_i)Q_i)$.

The fraction of the Earth covered at any instant is

$$\alpha_i = \begin{cases} (1-\beta_i)Q_i/\underline{Q}(h_i) & \text{if } (1-\beta_i)Q_i < \underline{Q}(h_i) \\ 1 & \text{if } (1-\beta_i)Q_i \geq \underline{Q}(h_i). \end{cases} \tag{20}$$

We calibrate the average beam angle ϕ so that the minimum covering numbers at the approximate mean altitudes of Starlink and OneWeb—550 km and 1,200km—are close to full coverage at their approximate current (Starlink, 3,351 satellites) and planned (OneWeb, 648 satellites) sizes. The value is listed in Table S1.

2.2.3 Latency

Consider the area that an operational satellite services as a circle of radius r_i centered at its vertical. Average signal latency is determined primarily by the average distance between consumers in the market and the satellite. Assuming that consumers are uniformly distributed over this area, the average distances are the lengths of the straight lines connecting them to the satellite.

From the Pythagorean theorem, the distance between a satellite at altitude h_i and a consumer located at a distance x of the center of the market is $\sqrt{x^2+h_i^2}$. Integrating over the radius of the market under a spatially-uniform consumer distribution, we obtain an average distance of

$$d_i = \int_0^{r_i} \frac{\sqrt{x^2+h_i^2}}{r_i} dx \tag{21}$$

$$= \frac{h_i}{2}\left[\sqrt{1+\left(\frac{r_i}{h_i}\right)^2} + \frac{h_i}{r_i} \ln\left(\frac{r_i}{h_i} + \sqrt{1+\left(\frac{r_i}{h_i}\right)^2}\right)\right], \tag{22}$$

where

$$r_i = \begin{cases} h_i \tan(\phi/2) & \text{if } (1-\beta_i)Q_i < \underline{Q}(h_i) \\ R/\sqrt{(1-\beta_i)Q_i} & \text{if } (1-\beta_i)Q_i \geq \underline{Q}(h_i). \end{cases} \tag{23}$$

If the signal travels at average speed v, we obtain the average time-of-flight for a single trip as d_i/v. Assuming that the signal makes λ trips and the electronic systems introduce a minimum latency of μ, the average latency for a constellation at altitude h_i and size Q_i is then

$$L_i = \lambda\ 10^3 d_i/v + \mu. \tag{24}$$

We calibrate λ and μ so that the implied latencies for the Starlink and OneWeb constellations are consistent with publicly available data on their service offerings [Ookla, 2022, Brodkin, 2019]. The parameter values are listed in Table S1. The implied latencies for Starlink and OneWeb are about 34 ms and 38 ms, respectively.

2.2.4 Bandwidth

An operational satellite in constellation i provides average bandwidth of κ Mb/s. Constellation i therefore supplies total bandwidth of $\kappa(1-\beta_i)Q_i$ shared among all covered subscribers. Given a coverage rate of α_i, only $\alpha_i D_i$ consumers can be active at the same time among the D_i subscribers to constellation i. Therefore the throughput at peak load for a subscriber receiving service will be equal to

$$S_i = \frac{\kappa(1-\beta_i)Q_i}{\alpha_i D_i}. \tag{25}$$

For tractability, we assume consumers anticipate bandwidth S_i per equation 25 under equilibrium demand (equations 12 and 13), thus taking the behavior of other consumers as given. Bandwidth for each constellation then simplifies to

$$S_L = \frac{3\kappa(1-\beta_L)Q_L}{2\alpha_L(2+\underline{\theta})N}, \tag{26}$$

$$S_F = \frac{3\kappa(1-\beta_F)Q_F}{\alpha_F(1-\underline{\theta})N}. \tag{27}$$

We calibrate the average bandwidth per satellite κ so that the implied peak-load throughput per subscriber for a constellation like Starlink (most satellites currently located near 550 km altitude, full coverage, 3,351 satellites and 1,000,000 subscribers) is consistent with publicly available data on Starlink [Ookla, 2022] for North America. The parameter value is listed in Table S1. The implied peak-load throughput per subscriber is about 84 Mb/s.

2.2.5 Satellite unit costs

The unit cost of a satellite includes the costs of manufacture, launch, and operations during lifetime, inclusive of ground stations. We let $C(h_i)$ be the annualized unit cost of a satellite. The annualized cost of a constellation of Q_i satellites is equal to $C(h_i)Q_i$.

Physical principles (e.g. the rocket equation) and empirical data support modeling the launch cost of a satellite as increasing in its altitude. There is less guidance on the operation cost during lifetime. We will approximate the annualized unit cost function as

$$C(h_i) = c - dh_i + \frac{e}{2}h_i^2, \tag{28}$$

where all parameters c, d and e are assumed positive. This specification implies that the annualized cost of a satellite is strictly convex and reaches a minimum at $\underline{h} = d/e$. We set the cost-minimizing altitude $\underline{h}$ to 500 km.

We calibrate the parameters of $C(\cdot)$ to match publicly available information about unit launch costs for the Starlink and OneWeb megaconstellations. Following Osoro and Oughton [2021] and public statements by SpaceX COO Gwynne Shotwell [Wang, 2019], we assume the cost of a reference satellite with a 5-year operational life is on the order of 500,000 \$, with additional operational and support infrastructure costs on the order of 125,000 \$. We thus approximate the annualized cost of a satellite at the cost-minimizing altitude $\underline{h}$ as 150,000 \$. Taken together, these figures imply that the annualized cost of building, launching, operating, and supporting a satellite at 550 km (Starlink) and at 1,200 km (OneWeb) altitudes are 152,500 \$. and 640,000 \$ respectively. The parameter values are listed in Table S2.

2.3 Avoidance maneuvers and service availability

To avoid collisions, satellites in constellation i must maneuver in response to at least some conjunctions. These maneuvers are an opportunity cost for operators, as they reduce their constellations' operational service time. To keep the model tractable and focus on economic behavior, we assume that all maneuvers are successful and result in no collisions.[5]

We assume that satellites will maneuver if and only if the conjunction is predicted to occur at or within a "maneuver safety margin" of ρ kilometers. As mentioned in the main text, this parameter may depend on technical, behavioral and regulatory factors, such as the constellations slotting architectures, the positional uncertainty in the object's trajectory [Arnas et al., 2021, D'Ambrosio et al., 2022], the operators' risk aversion and/or the implementation of avoidance guidelines coordinated internationally [Inter-Agency Debris Coordination Committee, 2017].[6]

Following prior literature [Talent, 1992, Lewis et al., 2009, Lifson et al., 2022], we model the probability $\delta(h)$ that any two satellites orbiting in a common shell at mean altitude h have a maneuver-inducing conjunction using kinetic gas theory as[7]

$$\delta(h) = \pi \frac{(2\rho)^2 \upsilon(h)}{V(h)}, \tag{29}$$

where

$$\upsilon(h) = \sqrt{\frac{GM}{R+h}} \tag{30}$$

is the velocity of an object in a circular orbit at altitude h, GM is the geocentric gravitational constant and R is the radius of the Earth, and

$$V(h) = \frac{4}{3}\pi \left((R+h+\Delta)^3 - (R+h-\Delta)^3 \right) \tag{31}$$

[5]Failed maneuvers would require explicit analysis of debris and collision risk dynamics—an important extension for future research.

[6]While in principle each object type could have a different safety margin (and even conjunction pair-specific safety margins), given the limited publicly available data on maneuver behavior we assume all objects use a common safety margin.

[7]Note that prior literature [Talent, 1992, Lewis et al., 2009, Lifson et al., 2022] calculates the probability of *collision*, as a function of the radii of the two objects, while we calculate the probability of *conjunction*, as a function of their maneuvering safety margins ρ.

is the volume of a circular shell of thickness 2Δ at altitude h above the Earth's surface. Satellites in different shells are assumed to have no conjunctions.

The *expected* number of maneuvers n_i that constellation i will have to perform per day is calculated by adding the number of conjunctions involving two satellites from constellation i (internal congestion) and one satellite from constellation i with any other objects in the same orbital shell (external congestion). Knowing that the constellation i operates Q_i satellites at altitude h_i, we obtain:

$$n_i = 24 \times 60 \times 60 \, \delta(h_i) \, \frac{Q_i \, (Q_i + Q_{-i})}{2} \tag{32}$$

where Q_{-i} represents the number of *other* objects in the same shell and the factor of $1/2$ reflects symmetric turn-taking behavior. We define the constellation system's daily *maneuver burden* as $n_L + n_F$.

We calibrate ρ to match open-source analysis of Starlink maneuver behaviors in Lewis [2022]. By the end of 2022, Lewis [2022] utilized reporting by SpaceX and publicly available data to infer that Starlink satellites were collectively conducting around 75 maneuvers per day. Data from USSPACECOM [2022] indicates that as of December 26th, 2022 there were approximately 5616 objects total (3015 of them Starlink satellites) within the 35 km shell centered at 550 km (the mean altitude of Starlink). This implies that ρ must solve $n_i = 75$, given $h_i = 550$, $Q_i = 3015$ and $Q_i + Q_{-i} = 5616$, giving a value of approximately $\rho = 0.150$ km. The value of ρ can be interpreted as follows: "if two satellites are predicted to approach within about 0.300 kilometers of each other then their operators commit to one of them maneuvering, each taking turns being the one to maneuver." Figure S2 gives a graphical representation of the numbers of maneuvers per day for a constellation at altitude h (x-axis) and and size Q (y-axis), considering only internal conjunctions. Note that since we predict close approaches using kinetic gas theory, our estimated safety margin may be smaller than those used in practice. This is because kinetic gas theory assumes objects move randomly, whereas in reality satellite trajectories are not random. Since we maintain this assumption throughout, the magnitudes of welfare gains from optimal orbit use will likely be unaffected.

Letting τ be the average operational time (hours) lost per avoidance maneuver which reduces service,[8] the fraction of operational time that constellation i loses to maneuvers ("congestion") is:

$$\beta_i = \frac{\tau \, n_i}{24 \, Q_i}. \tag{33}$$

In both the equilibrium and optimal solutions under the benchmark calibration, constellations are spaced far enough apart that there are no conjunctions between operational satellites in different constellations.

[8]Not all maneuvers necessarily reduce service time for the constellation as a whole. For example, the maneuver may involve satellites that are not transmitting or receiving signals and *a priori* not scheduled to do so till after the maneuver's duration. Similarly, the constellation may have a redundant satellite ready for that situation, or the maneuver may have been pre-planned with coverage arranged.

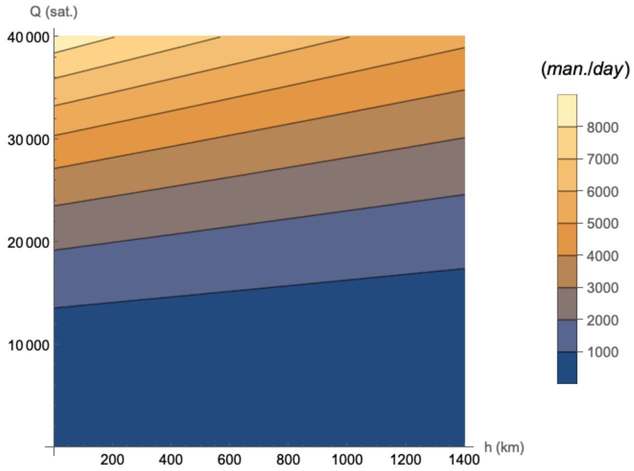

Figure S2: Number of maneuvers per day (internal conjunctions only). The contour map reflects that internal conjunctions increase in constellation size Q_i at an increasing rate as the maneuver distance ρ increases.

2.4 Solution concepts and algorithms

We apply two solution concepts: a "duopoly equilibrium" and an "optimal plan". The duopoly equilibrium predicts the allocation of orbital space if profit-maximizing firms compete in the two-stage game described in section 2.1 and below. The optimal plan predicts the allocation if constellations were designed by a benevolent planner to maximize global economic welfare from constellation telecommunications services.

We assume everywhere that the market is fully served, i.e. no consumers are left without telecom service. In equilibrium, this assumption is justified if it is profitable to serve all potential customers. In the optimum, this assumption is justified if it is socially beneficial to serve all consumers (i.e. equity of access). This assumption is satisfied in the equilibrium and optimum of the benchmark results presented in the main text.

2.4.1 Duopoly equilibrium

Firms first play a Stackelberg game of sequentially choosing altitude and constellation size. As described earlier, the Leader moves first in choosing an altitude and constellation size. In doing so they anticipate the Follower's optimal response in terms of altitude and constellation size as well as the equilibrium of the pricing subgame described in Section 2.1. The Follower best-responds to the Leader's choice anticipating only the price subgame equilibrium. Formally, using the solutions to the price equilibrium (equations 14 and 15), the Follower solves

$$\max_{h_F, Q_F} \pi_F = \frac{(1-\underline{\theta})^2}{9}(x_L - x_F)N - c(h_F)Q_F, . \tag{34}$$

taking (h_L, Q_L) as given, with x_L and x_F as defined in 16.

The solution to equation 34 gives the Follower's best-response functions, $h_F(h_L, Q_L)$ and $Q_F(h_L, Q_L)$. The Leader internalizes the Follower's behavior and solves

$$\max_{h_L, Q_L} \pi_L = \frac{(2+\underline{\theta})^2}{9}(x_L - x_F)N - c(h_L)Q_L, \tag{35}$$

anticipating $h_F(h_L, Q_L)$ and $Q_F(h_L, Q_L)$, with x_L and x_F as defined in 16.

Below, we restrict our attention to equilibria satisfying the following incentive compatibility constraints:

$$\underline{\theta} x_F - p_F \geq 0, \tag{36}$$

$$\pi_F \geq 0. \tag{37}$$

The first condition states that the consumer with the lowest valuation on telecom service gets non-negative utility from subscribing to the Follower's constellation. The second condition states that the Follower earns non-negative profits from staying in the market.

A duopoly equilibrium is a vector of choices for each firm, (h_L, Q_L, p_L) and (h_F, Q_F, p_F), and an indifferent consumer θ^*, such that no firm has an incentive to change their strategies, the indifferent consumer gains equal utility from either firm's service, all consumers have positive utility, and the Follower stays in the market (i.e. a joint solution to the system defined by 1, 5, 6, 34, 35, 36, and 37).

2.4.2 Social optimum

The planner seeks to maximize total economic welfare from constellation deployment, i.e. consumer surplus net of constellation production and environmental damage costs. To this end they may choose to deploy one or two constellations. We refer to these choices in the main text as "public utility constellations". A single very large constellation may be able to provide a high quality service to all consumers at a single low altitude (low latency and high bandwidth), but at the cost of more congestion and avoidance maneuvers. Two smaller constellations can provide differentiated services to better match varied consumer preferences at lower cost. We allow the planner to choose between welfare-maximizing one- and two-constellation architectures to determine the overall economically-optimal constellation design.

We assume environmental damage costs are proportional to the total number of satellites in orbit. The environmental damage cost of an additional satellite, denoted as f, captures various impacts throughout its lifecycle: damages from launch emissions and debris; collision risk, debris formation, and interference with astronomical observations [Adilov et al., 2015, Rao et al., 2020, Rouillon, 2020, Venkatesan et al., 2020, Massey et al., 2020]; contributions to climate change and ozone depletion during reentry; and potential damage to human and physical capital upon landing [Ryan et al., 2022, Byers et al., 2022]. Notably, these costs are externalities, overlooked by private constellation operators [Adilov et al., 2022, Lawrence et al., 2022]. With limited data on the full extent of damages from these externalities, further research is imperative to better understand and address these issues. We set the value of f to zero in our main scenarios and conduct sensitivity

analysis over the magnitude of damages to understand how they impact optimal and equilibrium orbital allocations.

When the planner allocates orbital space to one constellation, they solve

$$W_1 = \max_{h,Q} N \int_{\underline{\theta}}^{\underline{\theta}+1} \theta \, x \, \mathrm{d}\theta - \big(C(h) + f\big) Q. \tag{38}$$

When the planner allocates orbital space to two constellations, they solve

$$W_2 = \max_{h_L,Q_L,h_F,Q_F,\theta^*} N \int_{\theta^*}^{\underline{\theta}+1} \theta \, x_L \, \mathrm{d}\theta + N \int_{\underline{\theta}}^{\theta^*} \theta \, x_F \, \mathrm{d}\theta \tag{39}$$

$$- C(h_L) Q_L - C(h_F) Q_F - f(Q_L + Q_F). \tag{40}$$

Both problems are subject to all the physical constraints/functions as described earlier. The planner chooses the better of the two optimized constellation designs, solving

$$W = \max\{W_1, W_2\}. \tag{41}$$

We assume that the planner seeks to serve all consumers, so internalizes 36 as a constraint. However, the planner controlling two constellations has an additional degree of freedom: by letting the size of the constellation serving the lower end of the market shrink and moving the threshold θ^*, the two-constellation planner is able to reduce service to consumers with lower valuations. Thus, while the planner controlling one constellation is constrained to provide equitable service to all even if it reduces aggregate welfare, the planner controlling two constellations can segment the market as necessary to maximize aggregate welfare.

An optimal plan is a vector of constellation design characteristics ((h,Q) if $W_1 \geq W_2$ and (h_L, Q_L, h_F, Q_F) otherwise) which solves equation 41. If $W_2 > W_1$ then the optimal plan includes an indifferent consumer θ^* to solve equation 40.

2.4.3 Algorithms

To solve for the duopoly equilibrium and social optimum, we first generate a grid over possible values of (h_L, Q_L, h_F, Q_F).[9] We then compute the value of each constellation and total economic welfare with two constellations (i.e. the objective function being maximized in program 40) at each grid point. For the decentralized equilibrium, we then compute the Follower's profit-maximizing choices of (h_F, Q_F) conditional on the Leader's choices, i.e. we calculate the Follower's best response $(h_F(h_L, Q_L),\ Q_F(h_L, Q_L))$ for each choice (h_L, Q_L) the Leader could make, conditional on

[9]We describe the altitude grid construction in more detail in section 2.4.4.

the incentive compatibility conditions 36 and 37 being satisfied. Next, we compute the Leader's optimal choice of (h_L, Q_L) given the Follower's best response to that choice, and select the choice which maximizes the Leader's profits. Finally, firms' prices are set to solve the simultaneous-move game described in section 2.1, i.e. per equations 10 and 11. This is described more precisely in Algorithm 1.

Solving the planner's problem is more straightforward. We solve problems 38 and 40 using Generalized Simulated Annealing (GSA) [Tsallis and Stariolo, 1996] as implemented in the R package GenSA [Xiang et al., 2013], and select the one with the highest objective function value. This is the constellation design that maximizes economic welfare. In this setting GSA is preferable to methods such as Nelder-Mead or BFGS as the physical equations for service quality, latency, and coverage include non-differentiable points, and GSA provides better guarantees of finding the global optimum regardless of initial value. However, its runtime precludes use in the duopoly equilibrium problem where multiple nested GSA calls would be required. The objective surface for the planner's problem in the two-constellation case also features multiple solutions with near-identical welfare levels. To address instability in sensitivity analysis caused by multiple solutions with approximately equal welfare levels, we restrict the solver to focus on solutions where the constellation serving the upper end of the market is at a lower altitude than the one serving the lower end of the market. This has no effect on the solution in the benchmark calibration, and makes the solution paths for sensitivity analyses smoother without reducing economic welfare.

Algorithm 1: Algorithm to calculate decentralized equilibrium

1. Generate $grid(h_L, Q_L, h_F, Q_F)$.
2. Calculate $\pi_L, \pi_F, W, \underline{\theta} x_F - p_F$ at each grid point. Discard all points which don't satisfy conditions 36 and 37.
3. Calculate $\text{argmax}_{h_F, Q_F} \pi_F$ for each value of (h_L, Q_L). Call these (h_F^e, Q_F^e).
4. Calculate $\text{argmax}_{h_L, Q_L} \pi_L$ assuming Follower chooses (h_F^e, Q_F^e) in response. The profile $(h_L^e, Q_L^e, h_F^e, Q_F^e)$ is the Stackelberg equilibrium.
5. Set firms' prices according to equations 10 and 11 to reflect equilibrium in the simultaneous-move pricing subgame.

2.4.4 Orbital shell discretization

For tractability and following prior work, we discretize orbital space into a series of non-overlapping shells. For the duopoly equilibrium, we construct a grid of altitude values beginning at 200 km and ending at 900 km and use it as described in Algorithm 1. For the optimum, we constrain the GSA solver to a numerically-stable portion of the 200-900 km region identified through testing. For both the equilibrium and the optimal plan we apply external congestion only if the constellations are within 35 km of each other, consistent with the discretization used in Lifson et al. [2022] and the announced spacings of Starlink and Kuiper (which are set to be 50 km apart in the latest FCC filings).

2.5 Parameter values

Tables S1 and S2 lists the parameter values, parameter units, and a brief description of the calibration strategy where applicable. We set the number of consumers purchasing service, N, to 10,000,000 to reflect the assumption of a more-mature sector in the near future. Current LEO constellation subscribers globally are on the order of 1,000,000 [Jewett, 2022]. Note that we are not making a forecast of demand for constellation services from any particular operator, rather choosing a market size to reflect a particular scenario.

Parameter	Value	Description	Units	Source
**Physical constants**				
R	6371	Radius of the Earth.	$[km]$	
GM	3.986×10^5	Geocentric gravitational constant.	$[\frac{km^3}{s^2}]$	
v	3×10^5	Speed of light in vacuum.	$[\frac{km}{s}]$	
**Technical parameters**				
ϕ	0.4	Average beam angle.	$[radians]$	Calibrated to make minimum coverage numbers at 550 km and 1200 km altitudes consistent with Starlink and OneWeb sizes.
λ	2	Time-of-flight multiplier.	$[unitless]$	Reflects trips from satellite to ground station to consumer.
μ	30	Minimum hardware and network latency.	$[ms]$	Lower end of published latencies for Starlink and OneWeb.
κ	2.5×10^4	Peak throughput coefficient.	$[\frac{Mb/s}{satellite}]$	Calibrated to be consistent with average download speed for Starlink in North America near end of 2022 (≈1e7 subscribers).
τ	2	Average satellite service time lost per maneuver.	$[\frac{h}{maneuver}]$	Assumption.
ρ	0.150	Maneuver safety margin.	$[km]$	Calibrated to be consistent with analysis in Lewis [2022] and object count data as of 26-12-2022 from USSPACECOM [2022].
Δ	17.5	Orbital shell thickness.	$[km]$	Consistent with MOCAT altitude bin discretization used in Lifson et al. [2022].

Table S1: Table of calibrated physical and technical parameter values.

Parameter	Value	Description	Units	Source
**Economic parameters**				
a_L	9	Willingness-to-pay for lower latency.	$[\frac{\$/year}{ms}]$	From Liu et al. [2018]. Calibrated to match 6 $/year/ms WTP for lower latency.
a_S	3025	Willingness-to-pay for higher bandwidth.	$[\left(\frac{\$/year}{Mb/s}\right)^2]$	From Liu et al. [2018]. Calibrated to match 168 $/year WTP for bandwidth increase from 4 Mb/s to 10Mb/s and 288 $/year WTP for increase from 10 Mb/s to 25 Mb/s.
$\bar{L}$	275	Latency which makes consumers indifferent between status quo (no service) or a subscription with 25 ms latency and 75 Mb/s bandwidth for 1500 $/year	$[ms]$	
c	4×10^5	Annualized satellite unit cost.	$[\frac{\$/year}{satellite}]$	Calibrated to be consistent with Table 2 of Osoro and Oughton [2021] and public statements by Gwynne Shotwell [Wang, 2019].
d	1000	Annualized satellite unit cost / gross rate with altitude.	$[\frac{\$/year}{satellite-km}]$	Same as above, also calibrated to imply cost-minimizing altitude of 300 km.
e	2	Annualized satellite unit cost / gross rate with altitude squared.	$[\frac{\$/year}{satellite-km^2}]$	Same as above.
$\overline{\theta}$	0.5	Utility parameter for consumer with lowest WTP for satellite service.	$[unitless]$	Normalization.
N	10^7	Total number of targeted consumers.	$[people]$	Reflects mature market.

Table S2: Table of calibrated economic parameter values.

3 Sensitivity analysis

3.1 Market size and maneuver safety margin

This figure shows the monetary value of the welfare gain generated by optimal utility constellations as a function of target population size values. Figure S3 shows the dollar value of welfare

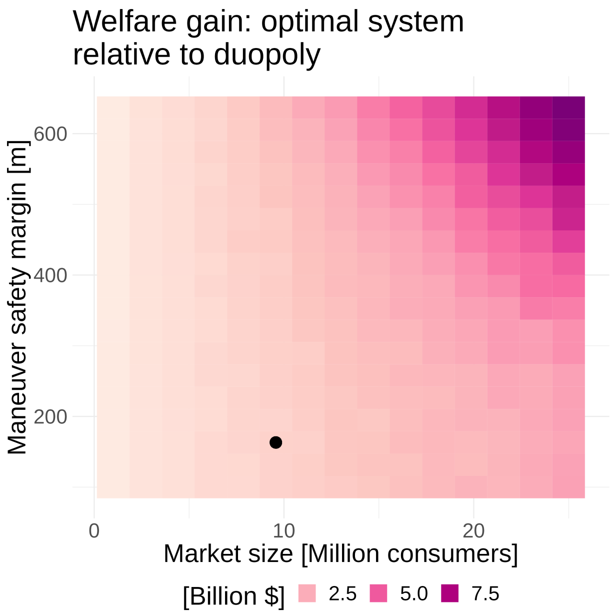

Figure S3: Dollar value gain in economic welfare from the economically-optimal two-constellation public utility system relative to the duopoly equilibrium across population size and maneuver safety margin. The black dot shows the benchmark calibration.

gain generated by the optimal public utility constellations over values of size of targeted population (N) and maneuver safety margin (ρ).

As mentioned in the main text, the monetary value of the gain from a two-constellation public utility system is increasing in N (holding ρ constant) even as the percentage gain decreases (main text Fig. 5). Figure S4 illustrates this point: holding ρ constant at the benchmark level and varying N, the welfare gain from moving from duopoly to a two-constellation system is roughly \$1 billion. At 20 million consumers, the gain is roughly \$2 billion.

3.2 Preference parameters

We conduct sensitivity analysis over preference parameters a_L, $\bar{L}$, and a_S, while maintaining the maximum and marginal willingness to pay for lower latency as in the benchmark calibration, given a standard internet service (25 ms latency, 75 Mb/s bandwidth available full time). The sensitivity analysis thus goes through the set of parameters satisfying these conditions:

- Same maximum WTP:

$$a_L(\bar{L} - L)\frac{S^2}{a_S + S^2} = 9(275 - L)\frac{S^2}{3025 + S^2} \tag{42}$$

- Same marginal WTP for latency improvements:

$$a_L\frac{S^2}{a_S + S^2} = 9\frac{S^2}{3025 + S^2} \tag{43}$$

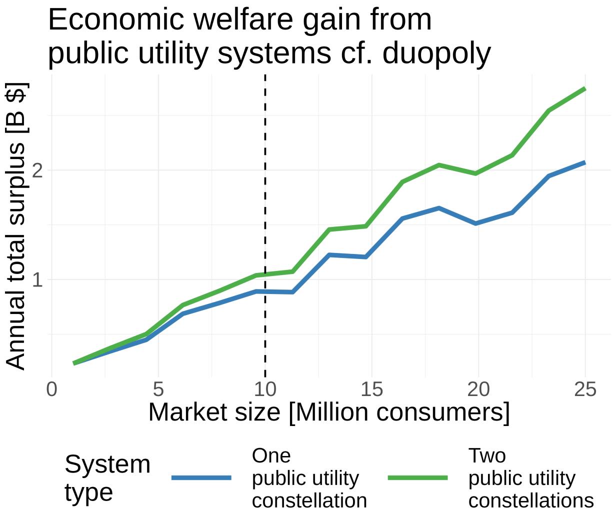

Figure S4: Dollar value gain in economic welfare from the economically-optimal two-constellation public utility system relative to the duopoly equilibrium across population size, holding maneuver safety margin constant. The dashed line shows the benchmark calibration.

when $L = 25$ and $S = 75$. This implies that:

$$\bar{L} = 275 \tag{44}$$

$$a_L = 9\frac{a_S + 5625}{8650}. \tag{45}$$

That is, in order to maintain the targets we set for maximum and marginal WTP, we must hold one parameter ($\bar{L}$) at the benchmark level and co-vary a_L and a_S as dictated by equation 45. We conduct the sensitivity analysis over $a_S \in [40^2, 70^2]$.

The total constellation sizes increase at a decreasing rate as a_L and a_S increase. The altitude also increases, though at a very slow rate. A higher preference for service quality (of which bandwidth is an important part) can justify decreased availability due to larger sizes at roughly the same altitude.

3.3 Environmental damages

We conduct sensitivity analysis over the magnitude of environmental damages, f, to identify how the optimal allocations change in response to these external costs. We find that total environmental damages on the order of $150,000 are sufficient to make the public utility constellation sizes match the duopoly sizes, though the orbital space allocations remain different. Figure 7 in the main text shows the total number of satellites and the threshold at roughly $150,000 in total environmental damages. Figure S6 shows the size and location allocations by constellation.

The single public utility constellation abruptly goes to zero at roughly $330,000 in total environmental damages. This is related to the equity constraint discussed in section 2.4.2, i.e. equation 36. The planner controlling a single constellation must provide equitable service to all consumers.

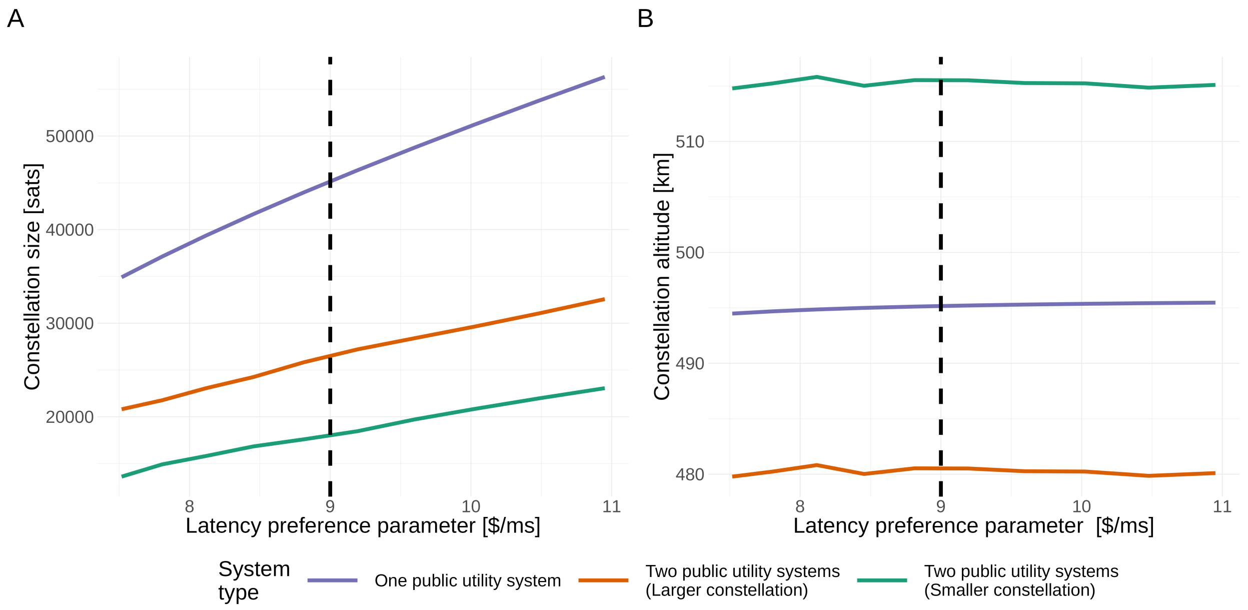

Figure S5: Change in number of satellites for public utility constellations as latency preference parameter a_L varies subject to equations 44 and 45. The dashed line shows the benchmark calibration.

When the environmental damages become high enough there is no way to provide such service while also generating surplus sufficient to cover the costs of the system. That is, the service quality becomes bad enough that consumers with a low valuation will prefer to not have it. The planner therefore ceases to provide constellation service once the environmental damages cross this threshold, since it is unable to provide all consumers with desirable service. The two-constellation planner is not similarly constrained. In this case, the planner ceases deployment of the smaller constellation once the $150,000 damages threshold is crossed, progressively reducing the size of the remaining system as well as the market it serves. This ensures that the system provides positive net benefits to the consumers it serves.

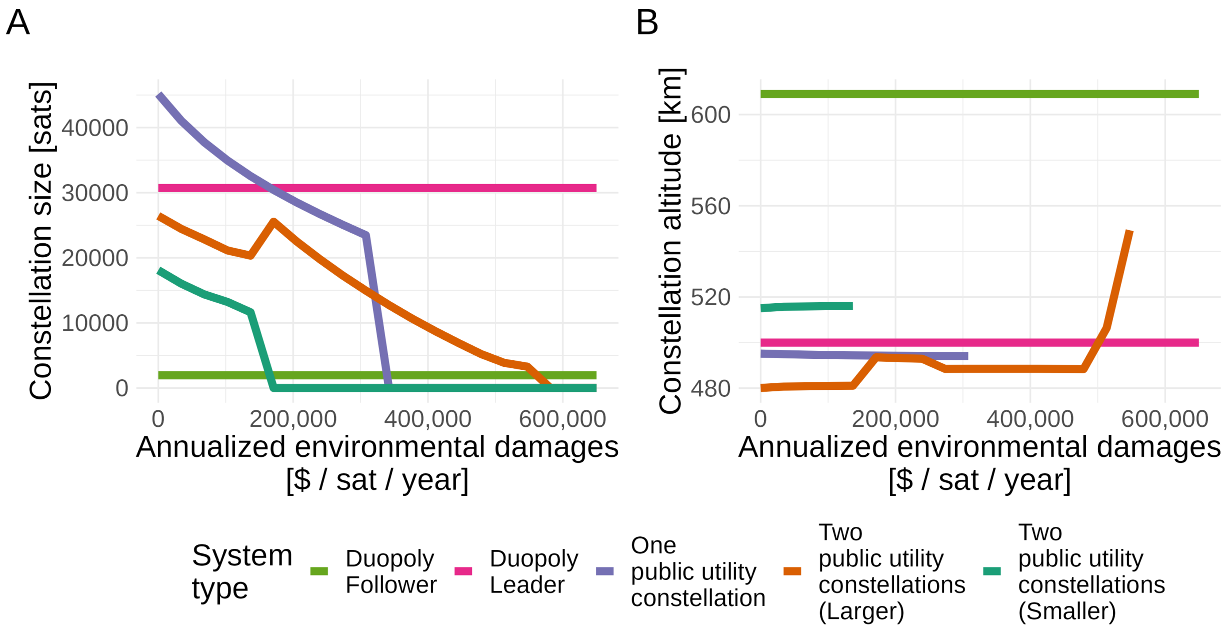

Figure S6: Change in total number of satellites for public utility constellations as total environmental damages from orbit use increase. In the benchmark calibration these damages are set to zero.

References

Edward C Prescott and Michael Visscher. Sequential location among firms with foresight. *The Bell Journal of Economics*, pages 378–393, 1977.

Imke De Pater and Jack J Lissauer. *Planetary sciences*. Cambridge University Press, 2015.

Ogutu B Osoro and Edward J Oughton. A techno-economic framework for satellite networks applied to low earth orbit constellations: Assessing starlink, oneweb and kuiper. *IEEE Access*, 9:141611–141625, 2021.

Yu-Hsin Liu, Jeffrey Prince, and Scott Wallsten. Distinguishing bandwidth and latency in households' willingness-to-pay for broadband internet speed. *Information Economics and Policy*, 45:1–15, 2018.

Ookla. Starlink slowed in Q2, competitors mounting challenges. https://www.ookla.com/articles/starlink-hughesnet-viasat-performance-q2-2022, 2022. Accessed: 2022-12-20.

Jon Brodkin. OneWeb's low-Earth satellites hit 400mbps and 32ms latency in new test. https://www.ookla.com/articles/starlink-hughesnet-viasat-performance-q2-2022, 2019. Posted: 2019-07-17. Accessed: 2022-12-20.

Brian Wang. SpaceX Starlink satellites could cost $250,000 each and Falcon 9 costs less than $30 million. https://www.nextbigfuture.com/2019/12/spacex-starlink-satellites-cost-well-below-500000-each-and-falcon-9-launches-less-tha html, 2019. Accessed: 2022-12-20.

David Arnas, Miles Lifson, Richard Linares, and Martín E Avendaño. Definition of low earth

orbit slotting architectures using 2d lattice flower constellations. *Advances in Space Research*, 67(11):3696–3711, 2021.

Andrea D'Ambrosio, Miles Lifson, Daniel Jang, Celina Pasiecznik, and Richard Linares. Projected orbital demand and leo environmental capacity. In *Advanced Maui Optical and Space Surveillance Technologies Conference Proceedings*, 2022.

Inter-Agency Debris Coordination Committee. IADC statement on large constellations of satellites in low-earth orbit. Technical report, 2017. Accessed April 29, 2023. Available from: https://www.iadc-home.org/documents_public/file_down/id/5253.

David L Talent. Analytic model for orbital debris environmental management. *Journal of spacecraft and rockets*, 29(4):508–513, 1992.

HG Lewis, GG Swinerd, RJ Newland, and A Saunders. The fast debris evolution model. *Advances in Space Research*, 44(5):568–578, 2009.

Miles Lifson, Andrea D'Ambrosio, David Arnas, and Richard Linares. How many satellites can we fit in low earth orbit?: Capacity integrating risk-based and intrinsic methods. In *Astrodynamics Specialist Conference*, 2022.

Hugh Lewis. "The reporting by SpaceX suggests that Starlink satellites (in total) are currently performing about 75 collision avoidance manoeuvres each day. with the simple extrapolation, the number could be $\tilde{1}$,000 per day by the end of 2027 [4/n]". https://twitter.com/ProfHughLewis/status/1610308012475834370, 2022. Posted: 2023-01-03. Accessed: 2023-01-04.

USSPACECOM. Space-Track.org. https://www.space-track.org, 2022. Accessed: 2022-12-26.

Nodir Adilov, Peter J Alexander, and Brendan M Cunningham. An economic analysis of earth orbit pollution. *Environmental and Resource Economics*, 60(1):81–98, 2015.

Akhil Rao, Matthew G Burgess, and Daniel Kaffine. Orbital-use fees could more than quadruple the value of the space industry. *Proceedings of the National Academy of Sciences*, 117(23): 12756–12762, 2020.

Sébastien Rouillon. A physico-economic model of low earth orbit management. *Environmental and Resource Economics*, 77(4):695–723, 2020.

Aparna Venkatesan, James Lowenthal, Parvathy Prem, and Monica Vidaurri. The impact of satellite constellations on space as an ancestral global commons. *Nature Astronomy*, 4(11): 1043–1048, 2020.

Robert Massey, Sara Lucatello, and Piero Benvenuti. The challenge of satellite megaconstellations. *Nature astronomy*, 4(11):1022–1023, 2020.

Robert G Ryan, Eloise A Marais, Chloe J Balhatchet, and Sebastian D Eastham. Impact of rocket launch and space debris air pollutant emissions on stratospheric ozone and global climate. *Earth's Future*, page e2021EF002612, 2022.

Michael Byers, Ewan Wright, Aaron Boley, and Cameron Byers. Unnecessary risks created by uncontrolled rocket reentries. *Nature Astronomy*, 6(9):1093–1097, 2022.

Nodir Adilov, Peter Alexander, and Brendan Cunningham. Understanding the economics of orbital pollution through the lens of terrestrial climate change. *Space Policy*, 59:101471, 2022.

Andy Lawrence, Meredith L Rawls, Moriba Jah, Aaron Boley, Federico Di Vruno, Simon Garrington, Michael Kramer, Samantha Lawler, James Lowenthal, Jonathan McDowell, et al. The case for space environmentalism. *Nature Astronomy*, 6(4):428–435, 2022.

Constantino Tsallis and Daniel A Stariolo. Generalized simulated annealing. *Physica A: Statistical Mechanics and its Applications*, 233(1-2):395–406, 1996.

Yang Xiang, Sylvain Gubian, Brian Suomela, and Julia Hoeng. Generalized simulated annealing for global optimization: the gensa package. *The R Journal*, 5(1):13, 2013.

Rachel Jewett. Spacex starlink internet service surpasses 1m subscribers. https://www.satellitetoday.com/broadband/2022/12/19/spacex-starlink-internet-service-surpasses-1m-subscribers/, 2022. Posted: 2022-12-19. Accessed: 2023-1-5.
